# *Notes from the Field: *Safety Monitoring of Novavax COVID-19 Vaccine Among Persons Aged ≥12 Years — United States, July 13, 2022–March 13, 2023

**DOI:** 10.15585/mmwr.mm7231a4

**Published:** 2023-08-04

**Authors:** Brittney Romanson, Pedro L. Moro, John R. Su, Paige Marquez, Narayan Nair, Brendan Day, Allison DeSantis, Tom T. Shimabukuro

**Affiliations:** ^1^Division of Healthcare Quality and Promotion, National Center for Emerging and Zoonotic Infectious Diseases, CDC; ^2^Office of Biostatistics and Pharmacovigilance, Center for Biologics Evaluation and Research, Food and Drug Administration, Silver Spring, Maryland; ^3^Immunization Services Division, National Center for Immunization and Respiratory Diseases, CDC.

The NVX-CoV2373 (Novavax) COVID-19 vaccine is a recombinant spike protein nanoparticle vaccine with Matrix-M adjuvant. Novavax is authorized and recommended as a primary 2-dose monovalent vaccination series in persons aged ≥12 years to prevent COVID-19 and as a monovalent booster dose in persons aged ≥18 years who are unable to or unwilling to receive an mRNA COVID-19 bivalent vaccine (*1*).

## Investigation and Outcomes

During July 13, 2022–March 13, 2023, a total of 69,227 Novavax doses were administered to persons aged ≥12 years in the United States, and 230 reports of adverse events (AEs) after Novavax vaccination were received by the Vaccine Adverse Event Reporting System (VAERS) (*2*). The median age of patients in the reports was 45 years (IQR = 31–61 years); 152 (66.1%) reports concerned females, and 104 (45.2%) concerned non-Hispanic White persons (Table). Within the study period, VAERS received no reports concerning pregnant women. Most VAERS reports (211; 91.7%) were classified as nonserious.* The most commonly reported AEs included dizziness (33; 14.3%), fatigue (26; 11.3%), and headache (25; 10.9%).

**TABLE:** Demographic characteristics in reports of adverse events after primary Novavax COVID-19 vaccination in persons aged ≥12 years* and most frequent Medical Dictionary for Regulatory Activities Preferred Terms^†^ in reports — Vaccine Adverse Event Reporting System, United States, July 13, 2022–March 13, 2023

Characteristic	Report classification, no. (%)		
	Serious^§^ (n = 19)	Nonserious (n = 211)	Total (N = 230)
**Sex**			
Female	12 (63.2)	140 (66.4)	**152 (66.1)**
Male	6 (31.6)	65 (30.8)	**71 (30.9)**
Unknown	<3 (5.3)	6 (2.8)	**7 (3.0)**
**Age group, yrs**			
12–17	0 (—)	5 (2.4)	**5 (2.2)**
18–49	8 (42.1)	111 (52.6)	**119 (51.7)**
50–64	6 (31.6)	58 (27.5)	**64 (27.8)**
≥65	5 (26.3)	37 (17.5)	**42 (18.3)**
**Race and ethnicity**			
American Indian or Alaska Native, NH	0 (—)	0 (—)	**0 (—)**
Asian, NH	<3 (5.3)	7 (3.3)	**8 (3.5)**
Asian, unknown ethnicity	0 (—)	<3 (0.5)	**<3 (0.4)**
Black or African American, NH	<3 (5.3)	8 (3.8)	**9 (3.9)**
Native Hawaiian or other Pacific Islander, NH	<3 (5.3)	0 (—)	**<3 (0.4)**
White, NH	12 (63.2)	92 (43.6)	**104 (45.2)**
White, unknown ethnicity	0 (—)	15 (7.1)	**15 (6.5)**
Hispanic or Latino**	0 (—)	24 (11.4)	**24 (10.4)**
Multiple races, NH	0 (—)	<3 (1.0)	**<3 (0.9)**
Unknown race, NH	0 (—)	<3 (1.0)	**<3 (0.9)**
Unknown race, unknown ethnicity	4 (21.1)	58 (27.5)	**62 (27.0)**
**MedDRA PT^††^**			
Chest pain	3 (15.8)	14 (6.6)	**17 (7.4)**
Dizziness	5 (26.3)	28 (13.3)	**33 (14.3)**
Fatigue	4 (21.1)	22 (10.4)	**26 (11.3)**
Fever	<3 (5.3)	15 (7.1)	**16 (7.0)**
Headache	3 (15.8)	22 (10.4)	**25 (10.9)**
Incorrect dose administered	0 (—)	16 (7.6)	**16 (7.0)**
Incorrect product formulation administered	0 (—)	16 (7.6)	**16 (7.0)**
Interchange of vaccine products	0 (—)	18 (8.5)	**18 (7.8)**
Nausea	3 (15.8)	16 (7.6)	**19 (8.3)**
Pain	0 (—)	19 (9.0)	**19 (8.3)**

**Abbreviations:** MedDRA PT = Medical Dictionary for Regulatory Activities Preferred Term; NH = non-Hispanic; VAERS = Vaccine Adverse Event Reporting System.

* Reports for persons aged ≥18 years were received by VAERS and had vaccination dates during July 13, 2022–March 13, 2023; reports for persons aged 12–17 years were received and had vaccination dates during August 19, 2022–March 13, 2023; includes reports with missing vaccination dates. When fewer than three reports were received within a category, the number is noted as “<3” to prevent inadvertent identification of the vaccine recipient.

^†^ https://www.meddra.org/how-to-use/basics/hierarchy

^§^ VAERS reports are classified as serious if any of the following are reported: hospitalization, prolongation of hospitalization, life-threatening illness, permanent disability, congenital anomaly or birth defect, or death. https://www.accessdata.fda.gov/scripts/cdrh/cfdocs/cfcfr/cfrsearch.cfm?fr

** Includes Hispanic or Latino ethnicity of unknown race.

^††^ Not mutually exclusive.

Among the 230 reports received, 19 (8.3%) were classified as serious; no deaths were reported after vaccination. Serious reports included one case of thrombosis, two of pericarditis, one of Guillain-Barré syndrome, and two of seizure; available medical records for these reports were reviewed. The remaining serious reports described chest pain, arrhythmia, sickness, hospitalization, adverse event not otherwise specified, balance disorder, peripheral neuropathy aggravated, and vaccine failure. The reports were primarily manufacturer reports with no records available for review. The report of thrombosis described a female with axillary-subclavian thrombosis occurring 6 days after vaccination; medical history suggested other potential causes (e.g., use of oral contraceptives). One of the two reports of pericarditis described a male aged 40–49 years with an abnormal electrocardiogram and simple circumferential pericardial effusion 3 days after vaccination; medical history suggested potential underlying causes (e.g., multiple previous SARS-CoV-2 infections, including with the Omicron B.1.1.529 variant). The second pericarditis report did not meet the CDC case definition for myocarditis or pericarditis (*3*). No records were available for the report of Guillain-Barré syndrome. Records were available for one report of seizure, which described new seizure onset in an adolescent who met the Brighton Collaboration case definition for seizure.^†^

Sixty-six (28.7%) reports were for vaccination errors, all classified as nonserious, including three (4.5%) documenting an AE (e.g., fever and fatigue). The most commonly reported vaccination error was administration of a (not yet authorized) booster dose of Novavax instead of an mRNA COVID-19 vaccine (21; 31.8%).

## Preliminary Conclusions and Recommendations

Although postauthorization safety data after receipt of a primary Novavax dose are limited by the low number of doses administered (0.01% of total COVID-19 vaccine doses administered) (*2*), available data are consistent with those from preauthorization clinical trials.^§^ No new safety concerns were identified. Limitations of this analysis include reporting biases and inconsistency in the quality and completeness of reports to VAERS (*4*). VAERS data generally cannot be used to determine whether a vaccine caused an adverse event. In addition, approximately one half of the reports representing adverse events of special interest lacked medical records for CDC review.

Vaccine administration errors are largely preventable with proper education and training. To help ensure proper vaccine administration, use of a prevaccination checklist is recommended.^¶^ In general, the same vaccine product is recommended for all doses of primary COVID-19 vaccination series, with certain exceptions (e.g., inability to receive an mRNA vaccine).** These measures can help persons stay up to date with recommended COVID-19 vaccinations, which will reduce illness and death from this serious disease.

## Footnotes

* VAERS reports are classified as serious if any of the following are reported: hospitalization, prolongation of hospitalization, life-threatening illness, permanent disability, congenital anomaly or birth defect, or death. https://www.accessdata.fda.gov/scripts/cdrh/cfdocs/cfcfr/cfrsearch.cfm?fr

†
https://brightoncollaboration.us/generalized-convulsion-case-definition-companion-guide

§
https://www.fda.gov/media/159897/download

¶
https://www.cdc.gov/vaccines/covid-19/downloads/pre-vaccination-screening-form.pdf

**
https://www.cdc.gov/vaccines/acip/recs/grade/covid-19-novavax-adult-booster-etr.html
